# Exogenous γ-aminobutyric acid promotes 2-acetyl-1-pyrroline biosynthesis by enriching glutamate and optimizing its metabolic flux in pumpkin

**DOI:** 10.3389/fpls.2026.1808004

**Published:** 2026-04-10

**Authors:** Jingjing Chang, Xuemei Zhu, Xiaoying Ye, Jing Li, Xiao Chen, Junxing Li, Baige Zhang

**Affiliations:** Key Laboratory for New Technology Research of Vegetable, Vegetable Research Institute, Guangdong Academy of Agricultural Science, Guangzhou, China

**Keywords:** 2-acetyl-1-pyrroline, betaine aldehyde dehydrogenase, glutamic acid, pumpkin, γ-aminobutyric acid

## Abstract

Aroma is a key quality trait determining the flavor and market competitiveness of pumpkins. Its characteristic fragrance mainly comes from 2-acetyl-1-pyrroline (2-AP), whose biosynthesis shares the common precursor with γ-aminobutyric acid (GABA) metabolic pathway. However, it remains unclear whether and how exogenous GABA regulates 2-AP synthesis in pumpkin. Given that the 2-AP biosynthetic pathway is highly conserved across various plant tissues, understanding its regulatory mechanisms in leaves provides a fundamental framework for improving fruit aroma quality. This study investigated the effects of exogenous GABA on 2-AP biosynthesis in pumpkin seedlings and elucidated the underlying regulatory mechanisms by analyzing key precursors, metabolic enzyme activities, and related gene expression. Foliar application of 500 mg L⁻¹ GABA significantly increased the 2-AP content in pumpkin leaves by 72.4%, and promoted seedling growth, with leaf area and fresh weight increasing by 57.7% and 58.4%, respectively. GABA treatment markedly elevated glutamate (Glu) content by 7.8-fold and reduced proline (Pro) levels by 13.6% at 30 h. Furthermore, GABA application enhanced the activities and gene expression of Δ¹-pyrroline-5-carboxylate synthase (P5CS) and proline dehydrogenase (ProDH), while suppressing those of betaine aldehyde dehydrogenase (BADH), thereby coordinately directing metabolic flux toward 2-AP synthesis. These findings demonstrate that exogenous GABA effectively promotes 2-AP biosynthesis in pumpkin by enriching the Glu pool and coordinately regulating key enzymes to suppress competitive metabolic pathways. This study provides a theoretical foundation for utilizing GABA as a biostimulant to improve aroma quality in pumpkin.

## 1 Introduction

Pumpkin (*Cucurbita moschata* Duchesne ex Poiret.) is a widely cultivated and economically important cucurbit vegetable in China, recognized for its large cultivation scale, high yield, and rich nutritional composition (Paris and Brown, 2005; Kulczyński et al., 2020). Among various cultivars, the “Xiangyu” pumpkin has gained considerable consumer preference due to its distinctive aroma and enhanced nutritional value (Junxing et al., 2022). Research has shown that 2-Acetyl-1-pyrroline (2-AP) has been identified as the principal flavor-aroma compound in “Xiangyu” pumpkin, with its concentration strongly correlated with aroma intensity and sensory quality (Deng et al., 2023). Previous studies have reported significant 2-AP accumulation in both the fruits and leaves of aromatic pumpkin cultivars (Li et al., 2019; Zhao et al., 2025). Moreover, the core biosynthetic pathway of 2-AP is highly conserved across diverse plant tissues (Imran et al., 2023). This metabolic conservation suggests that the regulatory patterns and precursor mobilization established during the vegetative stage serve as a physiological foundation that determines the overall aromatic potential of the plant. Consequently, clarifying the regulatory effects of exogenous biostimulants on seedling leaves not only provides fundamental mechanistic insights into aroma modulation but also contributes to the development of targeted strategies for enhancing 2-AP accumulation and the flavor quality of mature fruits.

2-AP, identified as the key contributor to the characteristic aroma of fragrant rice (Wakte et al., 2017), is an important aromatic compound characterized by high heritability and widespread occurrence in various plant species, including fragrant rice, coconut, mung bean, muskmelon, and pumpkin (Adams and De Kimpe, 2006; Attar et al., 2017; Dumhai et al., 2019; Okpala et al., 2019). The accumulation of 2-AP in crops is determined not only by genetic background but is also significantly influenced by external environmental factors and cultivation practices, such as nutrient application, environmental conditions, farming methods, and treatment with exogenous plant growth regulators (Luo et al., 2022; Imran et al., 2023; Zhang et al., 2023). Among environmental factors, relatively low temperatures during the grain-filling stage have been shown to promote 2-AP biosynthesis in rice (Kong et al., 2020). Additionally, specific nutrients such as silicon and zinc application can significantly enhance 2-AP content in fragrant rice grains (Mo et al., 2017; Luo et al., 2019). Beyond these environmental and nutritional influences, plant growth regulators have emerged as a more direct and manipulable strategy for improving crop quality. For instance, foliar application of paclobutrazol has been reported to enhance aroma, grain quality, and yield in fragrant rice (Xing et al., 2023). The effect of 6-benzylaminoadenine (6-BA) treatment on 2-AP accumulation in fragrant rice is concentration-dependent, that is, low concentration (15 mg L⁻¹) could significantly promote 2-AP accumulation, while high concentration (30 mg L⁻¹) inhibited its synthesis (Wang et al., 2015).

The biosynthetic pathway of 2-AP is relatively complex and involves multiple key enzymes, including proline dehydrogenase (ProDH), ornithine aminotransferase (OAT), Δ¹-pyrroline-5-carboxylate synthase (P5CS), and betaine aldehyde dehydrogenase (BADH). The primary precursors for its synthesis include proline (Pro), glutamate (Glu), and ornithine (Orn). These precursors are converted into pyrroline-5-carboxylate (P5C) under the catalysis of ProDH, P5CS, and OAT, respectively. P5C is then decarboxylated to form Δ¹-pyrroline, which subsequently serves as the direct precursor for 2-AP synthesis (Hien et al., 2003; Wakte et al., 2017). Additionally, ornithine can be metabolized through multiple enzymatic reactions to produce γ-aminobutyraldehyde (GABald), which subsequently cyclizes to form Δ¹-pyrroline (Luo et al., 2021b). Studies have demonstrated that exogenous application of appropriate concentrations of amino acids can effectively promote 2-AP accumulation (Luo et al., 2020; Luo et al., 2020; Huang et al., 2023).

GABald functions not only as a critical intermediate in 2-AP biosynthesis but also as a precursor for γ-aminobutyric acid (GABA) synthesis. Under the catalysis of BADH, GABald can be converted into GABA, a process that competitively reduces the carbon flux toward 2-AP formation (Mo et al., 2015; Luo et al., 2021a). Genetic editing of the BADH2 gene to reduce or abolish BADH enzyme activity has been shown to suppress the conversion of GABald to GABA, thereby promoting 2-AP accumulation (Renuka et al., 2022). For instance, in rice, an 8-bp deletion in the *OsBADH2* gene results in reduced enzyme activity and consequently leads to the aromatic phenotype (Giang et al., 2023). Similarly, knockout of *GmBADH2* in soybean enables the synthesis of 2-AP and imparts aroma in originally non-aromatic varieties (Arikit et al., 2010).

GABA, a significant non-protein amino acid, plays crucial roles in plant growth, development, stress responses, and the regulation of agricultural product quality. Exogenous GABA application has been shown to improve tomato fruit quality by modulating the metabolism of amino acids, organic acids, and sugars (Wu et al., 2024). Notably, appropriate GABA treatment enhanced 2-AP content in rice by 6.22%–19.44% (Xie et al., 2020). Under nitrogen application conditions, GABA treatment increased 2-AP content in multiple rice cultivars by enhancing the activity of key biosynthetic enzymes (Xie et al., 2019). Although direct evidence for GABA-mediated regulation of 2-AP synthesis remains limited, their metabolic linkage provides a robust theoretical premise for this investigation. Because GABA and 2-AP compete for a common biosynthetic pathway and the critical intermediate γ-aminobutyraldehyde (GABald), we hypothesize that exogenous GABA application may induce feedback regulation. This process could suppress the conversion of GABald into endogenous GABA, consequently redirecting the metabolic carbon flux toward Δ¹-pyrroline synthesis and ultimately promoting 2-AP accumulation. Given the important role of GABA in the 2-AP biosynthesis pathway, this study investigated the effects of exogenous GABA on 2-AP biosynthesis in “Xiangyu” pumpkin and elucidated the underlying regulatory mechanisms at physiological and molecular levels. The findings provide new insights into the GABA-mediated formation of aromatic compounds in plants and establish a theoretical foundation for targeted regulation of aroma quality in pumpkin using growth regulators, thereby enhancing its economic value.

## 2 Materials and methods

### 2.1 Experimental materials and treatments

The test material was the Xiangyu pumpkin cultivar ‘Xiangyuanzao’, provided by the Vegetable Research Institute of the Guangdong Academy of Agricultural Sciences. Pumpkin seeds were sterilized, soaked in distilled water for 6 h, and then germinated in the dark in a constant temperature incubator at 30 °C. Germinated seeds were sown in nutrient pots filled with a substrate mixture (peat:vermiculite = 3:1, v/v) and grown in a controlled climate chamber under the following conditions: a 14/10-h photoperiod, a temperature of 28 °C/18 °C, and 85% relative humidity. The experiment was conducted using three-leaf stage seedlings.

The objective of Experiment 1 was to screen for the GABA concentration that enhanced 2-AP content in pumpkin. Seedlings were foliar sprayed with GABA solutions at concentrations of 0, 150, 250, 500, and 800 mg L⁻¹. At 36 h after GABA treatment, the third true leaf from the base was collected for 2-AP content determination. 500 mg L⁻¹ GABA was selected for subsequent experiments.

The objective of Experiment 2 was to investigate the regulatory effect of GABA on 2-AP synthesis in pumpkin. This experiment included two treatments: 0 (CK, control) and 500 mg L⁻¹ GABA (GABA treatment). On the 10th day of treatment, biomass, plant height, stem diameter, leaf area, and chlorophyll content were measured. The third true leaf from the base was sampled at 12, 18, 24, 30, 36, and 48 h after GABA treatment, immediately frozen in liquid nitrogen, and stored at −80 °C for subsequent analysis. Three biological replicates were used for each treatment, with each replicate representing one individual seedling grown in a single pot.

### 2.2 Measurement of growth parameters and chlorophyll content

Plant height and stem height of pumpkin seedlings were measured using a ruler and expressed in centimeters (cm). Stem diameter was determined using a vernier caliper and reported in millimeters (mm). The total leaf area per seedling was measured using Microtek Scanner (ScanMaker i800 Plus, China) and expressed in square millimeters (mm²). Fresh weight (FW) was measured immediately after sampling using a balance with 0.001 g precision. For dry weight (DW), plant samples were first deactivated at 105 °C for 30 min and then dried at 75 °C until a constant weight was achieved, after which the DW was recorded. Both FW and DW are expressed in grams (g).

Chlorophyll content was extracted and quantified following the method described by Arnon (1949) with minor modifications. Leaf discs of uniform size were obtained from pumpkin leaves using a 0.2 cm diameter punch. A 0.15 g sample of leaf discs was placed in a 25 mL test tube, to which 20 mL of 95% ethanol was added. The extraction was performed in the dark at room temperature until the leaf tissue turned white. The final volume was then adjusted to 25 mL with 95% ethanol. The absorbance of the extract was measured at 665 nm, 649 nm, and 470 nm using a microplate spectrophotometer (Epoch, BioTek, South Burlington, VT, USA).

### 2.3 2-AP content determination

2-AP content was analyzed using headspace solid-phase microextraction coupled with gas chromatography-mass spectrometry (HS-SPME/GC-MS) (Li et al., 2019). Following sampling, leaf tissues were immediately frozen in liquid nitrogen and subsequently freeze-dried at −50 °C (CTFD-18S-U Freeze Dryer, CREATRUST Co., Ltd.). The dried samples were ground into a fine powder for 2-AP extraction and detection.

For analysis, 0.3000 g of the powdered sample was placed in a 20 mL headspace vial, and 1 μL of 2, 4, 6-trimethylpyridine (TMP) in n-hexane (250 ng μL⁻¹) was added as an internal standard. The vial was immediately sealed and placed in a 70 °C water bath. A DVB/Carbon WR/PDMS Smart SPME Arrow fiber (Agilent, USA) was then inserted into the headspace of the vial. After equilibrating for 2 min, the extraction was conducted at 70 °C for 15 min. The SPME fiber was subsequently injected into the GC inlet for thermal desorption at 250 °C for 2 min.

GC conditions: gas chromatography column was an HP-5MS UI capillary column (30 m × 0.25 mm, 0.25 μm), carrier gas was helium, the flow rate was 1.0 mL/min, and the inlet temperature was maintained at 250 °C. The temperature program was as follows: initial temperature 35 °C held for 2 min, ramped at 4 °C/min to 170 °C (no hold), then increased at 20 °C/min to 250 °C (no hold). MS conditions: detection was carried out using an electron impact (EI) ion source operated at 70 eV. The ion source temperature was set at 230 °C, and the quadrupole temperature at 150 °C. Data acquisition was performed in full scan mode with a mass range of m/z 35–300. The peak areas of the 2-AP and internal standard quantitation ion peaks were extracted using Agilent Masshunter Quantitative Analysis (Version 10.0).

### 2.4 Determination of Pro, Glu, Orn, and GABA

Pro content was determined according to the ninhydrin method described by Bao et al (Bao et al., 2018), with absorbance measured at 520 nm, and results were expressed in micrograms per gram fresh weight (μg g⁻¹ FW). Orn content was quantified following the method of Huang et al (Huang et al., 2005).

Ninhydrin (25 mg·mL⁻¹) was dissolved in a mixed acid solution consisting of 6 mol L⁻¹ phosphoric acid (H_3_PO_4_) and glacial acetic acid at a volume ratio of 1:3, which was used as the coloring reagent. Following addition of sample, the mixture was reacted in a 100 °C water bath for 60 min. After cooling to room temperature, the L-ornithine content was quantified colorimetrically by measuring absorbance at 510 nm. Glu and GABA contents were analyzed using colorimetric assay kits (Guangzhou Hongshuolin Bio-technology Co., Ltd., China), following the manufacturer’s instructions.

### 2.5 Enzyme activity assays

P5CS activity was determined according to Zhang and Lu (Zhang et al., 1995). Fresh tissue (0.1 g) was homogenized in 50 mmol L⁻¹ Tris-HCl buffer (pH 7.5) containing 7.0 mmol L⁻¹ MgCl_2_, 1.0 mmol L⁻¹ KCl, 3.0 mmol L⁻¹ EDTA, 1.0 mmol L⁻¹ DTT, and 5% (w/v) polyvinylpyrrolidone (PVP). The reaction mixture consisted of 50 mmol L⁻¹ Tris-HCl buffer (pH 7.0), 20 mmol L⁻¹ MgCl_2_, 50 mmol L⁻¹ sodium glutamate, 10 mmol L⁻¹ ATP, 100 mmol L⁻¹ hydroxylamine, and enzyme extract. After incubation at 37 °C for 5 min, the reaction was terminated by adding 2.5 mol L⁻¹ HCl solution containing 2.5% FeCl_3_ and 6% trichloroacetic acid (TCA). Absorbance was measured at 340 nm, and enzyme activity was expressed as μmol min⁻¹ g⁻¹ FW.

OAT activity was assayed following the method of Luo et al (Luo et al., 2021b). Samples (0.1 g) were homogenized in 1 mL of 50 mmol L⁻¹ phosphate buffer (pH 8.0) containing 1 mmol L⁻¹ dithiothreitol. The homogenate was centrifuged at 12,000 r min⁻¹ for 10 min at 4 °C to obtain the enzyme extract. The reaction system contained 50 mmol L⁻¹ phosphate buffer (pH 8.0), 35 mmol L⁻¹ L-ornithine, 5 mmol L⁻¹ α-ketoglutarate, 0.05 mmol L⁻¹ pyridoxal phosphate, and 0.1 mL of enzyme extract. After 20 min of reaction at 25 °C, the process was stopped by adding 3 mol L⁻¹ perchloric acid. Followed by addition of 2% ninhydrin solution the mixture was heated in a boiling water bath for 20 min. After cooling and centrifugation, the supernatant was discarded, and the precipitate was dissolved in absolute ethanol. Absorbance was measured at 510 nm. OAT activity was expressed as μmol min⁻¹ g⁻¹ FW.

ProDH and glutamate decarboxylase (GAD) activities were measured using commercial assay kits (Guangzhou Hongshuolin Bio-technology Co., Ltd., China) following the manufacturer’s protocols. Δ¹-Pyrroline-5-carboxylate reductase (P5CR) and BADH activities were determined using ELISA kits (Shanghai Enzyme-linked Biotechnology Co., Ltd., China) according to the supplied instructions. All enzyme activities were expressed as μmol min⁻¹ g⁻¹ FW.

### 2.6 RNA extraction and RT-qPCR analysis

Total RNA was extracted from pumpkin leaves using the RNA simple Total RNA Kit (Tiangen Biotech), following the manufacturer’s instructions. After quality control, the total RNA was used for synthesizing the first-strand cDNA with FastKing gDNA Dispelling RT SuperMix (Tiangen Biotech) according to the manufacturer’s protocol. Real-time quantitative PCR (RT-qPCR) was performed using ArtiCan™ SYBR qPCR Mix (Tsingske Biotech) on a CFX96 Real-Time PCR Detection System (Bio-Rad, Hercules, CA, USA). *Actin* (Gene ID: *CmoCh11G016220*) was used as the internal reference gene. Relative gene expression levels were calculated using the 2^−ΔΔCT^ method (Livak and Schmittgen, 2001). Gene-specific primers were designed using Primer Premier 6 and listed in **Supplementary Table 1**.

### 2.7 Statistical analysis

The experiment was arranged in a completely randomized design with three independent biological replicates per treatment. Data are presented as the mean ± standard deviation (SD) of three replicates. Statistical analysis was performed using one-way analysis of variance (ANOVA), followed by Duncan’s multiple range test for multiple comparisons or independent samples t-test for comparisons between two groups, to determine significant differences among treatments. All figures were generated using Origin 2021 (OriginLab Corporation, Northampton, MA, USA).

## 3 Result

### 3.1 Concentration-dependent effect of exogenous GABA on 2-AP accumulation in pumpkin leaves

**Figure 1:**
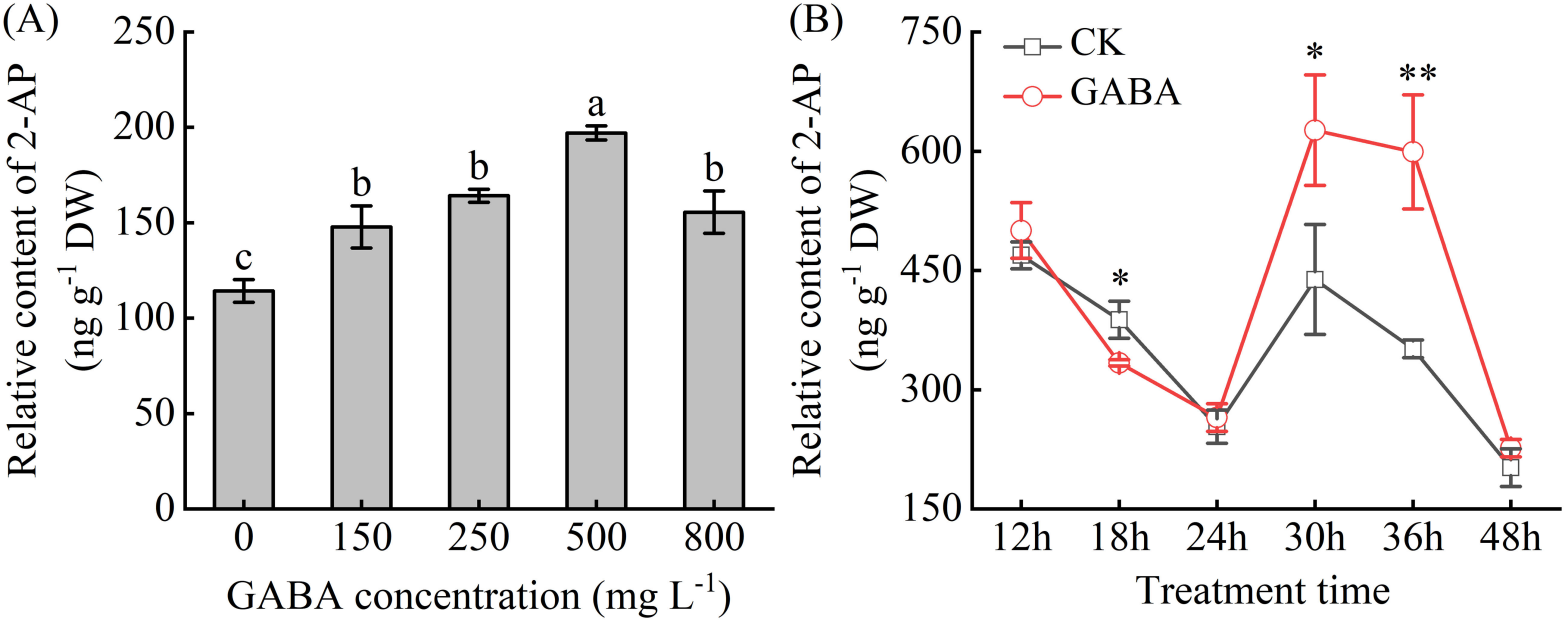
Effect of γ-aminobutyric acid (GABA) on 2-acetyl-1-pyrroline (2-AP) content in leaves of “Xiangyuanzao” pumpkin. **(A)** Effect of different concentrations of GABA on 2-AP content. Different letters indicate significant differences among treatments at *P* < 0.05 according to Duncan’s test. **(B)** Temporal dynamics of 2-AP content after GABA treatment. * and ** denote significant differences from CK at *P* < 0.05 and *P* < 0.01, respectively. CK, control; GABA, γ-aminobutyric acid.

Exogenous GABA treatment significantly increased the 2-AP content in pumpkin leaves in a concentration-dependent manner. The 2-AP content showed an initial increase followed by a decrease with rising GABA concentrations (**Figure 1A**). Compared with CK, the leaves treated with 150, 250, 500, and 800 mg L⁻¹ GABA exhibited increases in 2-AP content by 29.32%, 43.61%, 72.42%, and 36.05%, respectively (**Figure 1A**). The most pronounced effect was observed at 500 mg L⁻¹. However, the content of 2-AP in the leaves of pumpkin plants treated with 800 mg L⁻¹ GABA decreased by 21.09% compared with that of plants treated with 500 mg L⁻¹ GABA. Therefore, 500 mg L⁻¹ was identified as the optimal GABA concentration for enhancing 2-AP accumulation in pumpkin seedlings and was selected for subsequent experiments.

Regarding the temporal dynamics, the 2-AP content exhibited a general pattern of decrease, followed by an increase and subsequent decline during the 12 to 48 h after GABA treatment, peaking at 30 h (**Figure 1B**). Although the overall temporal trend remained unchanged between CK and GABA treatment, exogenous GABA significantly elevated the accumulation of 2-AP. Notably, at 30 and 36 h, the 2-AP content was 42.83% and 70.52% higher than that in CK, respectively, indicating a substantial promotive effect of GABA on 2-AP biosynthesis.

### 3.2 GABA enhanced growth of pumpkin seedlings

**Figure 2:**
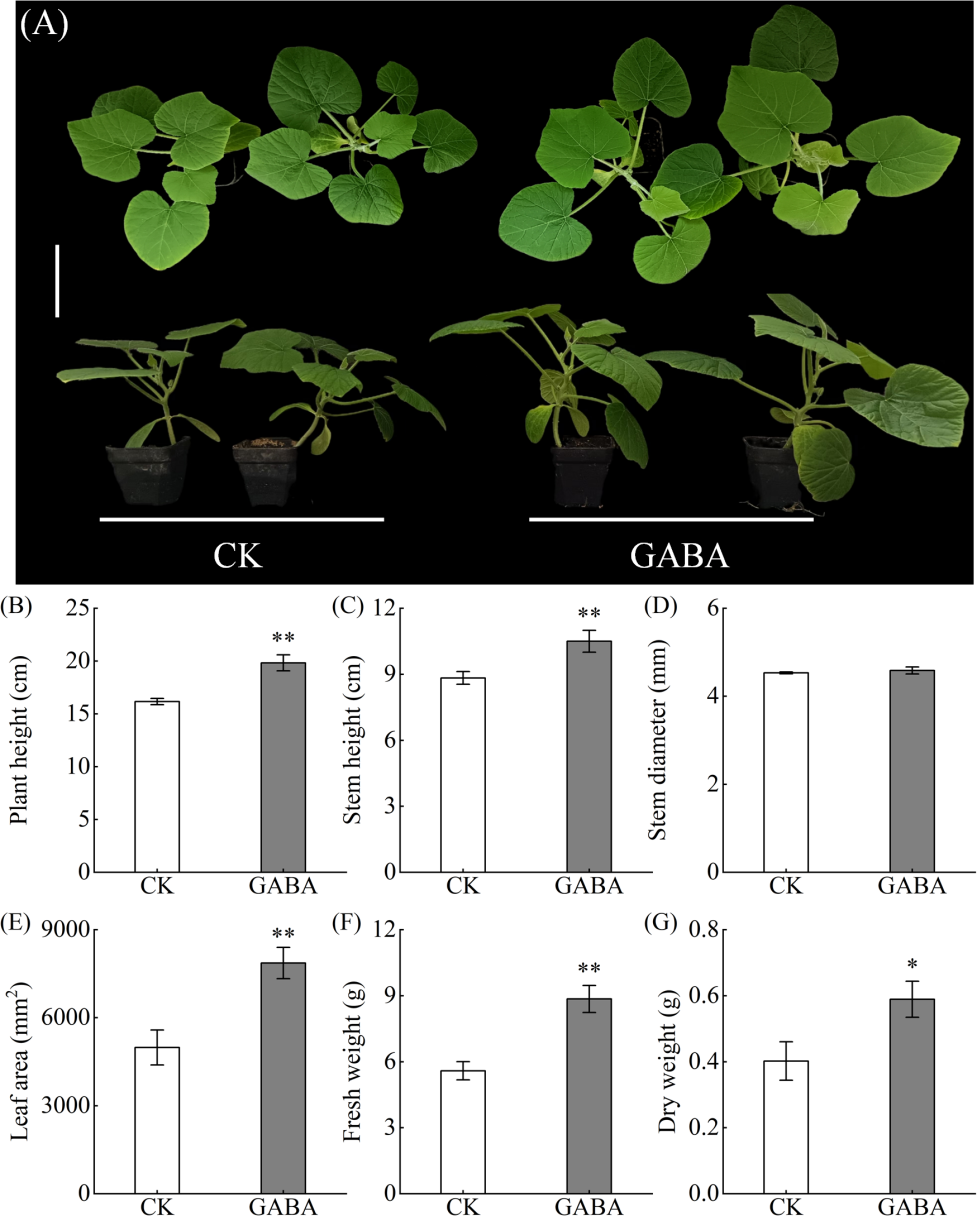
Effects of GABA on the growth of “Xiangyuanzao” pumpkin seedlings. **(A)** Phenotypic diagram; bar = 10cm; **(B–G)** Growth indicators. Data are presented as the mean ± standard deviation (SD) of three replicates. and * represent significant differences at the levels of P < 0.05 and P < 0.01, respectively. CK, control; GABA, ɣ-aminobutyric acid.

Exogenous GABA application significantly promoted the growth of pumpkin seedlings (**Figure 2**). Compared with CK, GABA treatment enhanced morphological parameters including plant height, stem height, and leaf area (**Figure 2B**). On the 10th day of treatment, plant height, stem height, and leaf area were significantly increased by 22.68%, 18.87%, and 57.69%, respectively, indicating a positive role of GABA in seedling morphogenesis. Dry matter accumulation, a key indicator of organic synthesis and storage capacity in plants, was markedly influenced by GABA treatment. Both fresh and dry weights of GABA-treated seedlings were significantly higher than those of CK, with increases of 58.38% and 46.49%, respectively. Regarding photosynthetic pigments, GABA treatment had a moderate influence on the contents of chlorophyll a, chlorophyll b, and carotenoid (**Supplementary Figure 1**). Chlorophyll a and chlorophyll b levels increased by 2.29% and 3.42%, respectively, whereas carotenoid content decreased by 5.17%. However, none of these changes reached statistical significance. In summary, exogenous GABA primarily improved pumpkin seedling growth by promoting morphological development and increasing biomass accumulation.

### 3.3 GABA modulated the accumulation of key precursors in the 2-AP biosynthetic pathway

**Figure 3:**
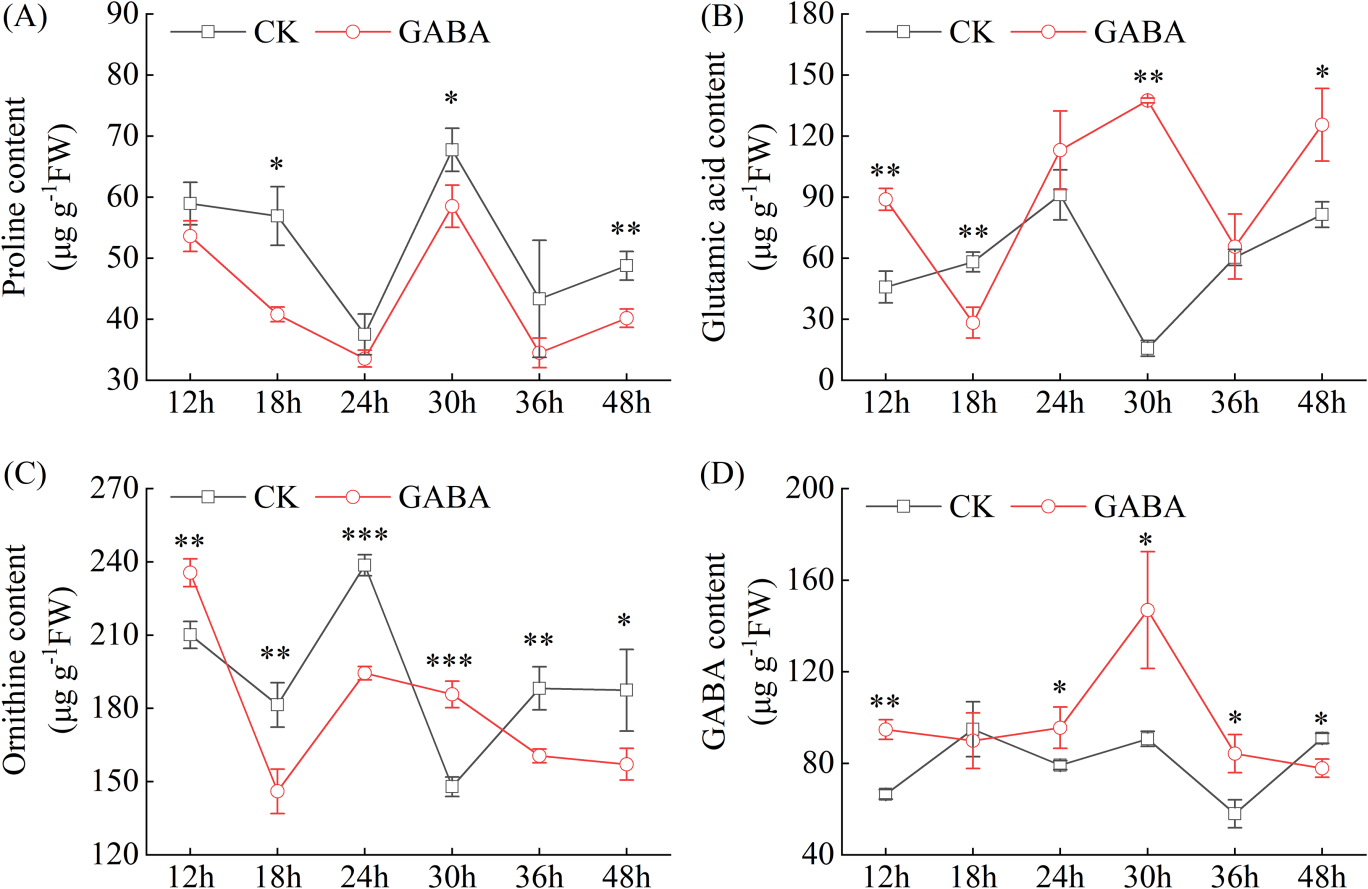
Effects of GABA on the contents of proline **(A)**, glutamate **(B)**, ornithine **(C)**, and GABA **(D)** in pumpkin seedling leaves. Data are presented as the mean ± standard deviation (SD) of three replicates. * and ** indicate significant differences from CK at *P* < 0.05 and *P* < 0.01, respectively. CK, control; GABA, γ-aminobutyric acid.

Pro, Glu, and Orn serve as key precursors in the 2-AP biosynthetic pathway. Foliar application of GABA significantly altered the accumulation dynamics of these metabolites in pumpkin leaves (**Figure 3**). Pro content exhibited a fluctuating pattern, reaching its peak at 30 h. While GABA treatment did not alter this overall trend, it significantly suppressed Pro accumulation, with notable reductions of 28.31%, 13.63%, and 17.59% observed at 18 h, 30 h, and 48 h, respectively, relative to CK (**Figure 3A**). In contrast, GABA treatment markedly altered the accumulation pattern of Glu. With the exception of the 18 h, GABA application significantly increased Glu content at all other sampling times. The most pronounced effect was recorded at 30 h, where Glu levels in GABA-treated leaves were 7.8-fold higher than those in CK (**Figure 3B**). Additionally, Glu content in GABA-treated leaves significantly increased by 93.98% and 54.17% at 12 h and 48 h, respectively, compared to CK. GABA treatment did not alter the overall temporal dynamics of Orn content, but it significantly modulated its accumulation levels at specific time points (**Figure 3C**). Compared with the CK, Orn content increased by 12.11% and 25.57% at 12 h and 30 h after GABA treatment, respectively. In contrast, it decreased by 19.51%, 18.52%, 14.70%, and 16.18% at 18 h, 24 h, 36 h, and 48 h, respectively. Furthermore, exogenous GABA application elevated endogenous GABA levels, which peaked at 30 h with a 62.02% increase relative to CK (**Figure 3D**). Notably, this significant accumulation of endogenous GABA was consistent with the substantial accumulation of Glu at 30 h, despite their overall temporal trajectories diverging within 48 h. In summary, exogenous GABA redirected the metabolic flux of 2-AP precursors in pumpkin leaves, primarily by significantly increasing the pools of Glu and endogenous GABA, while reducing the levels of Pro and Orn.

### 3.4 Effect of GABA on the activities of key enzymes in the 2-AP biosynthetic pathway

**Figure 4:**
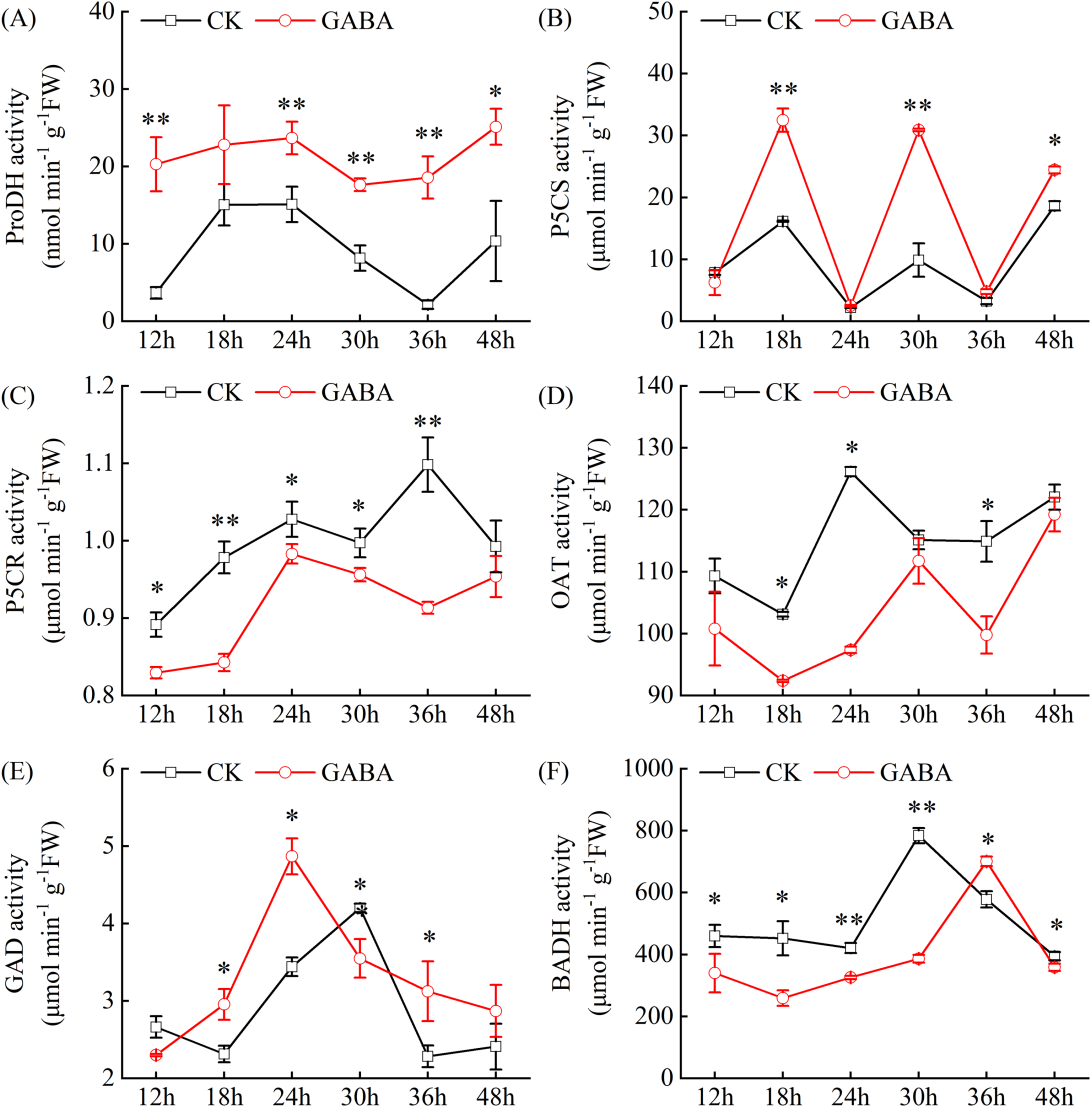
Effects of GABA on the activities of key enzymes involved in 2-AP biosynthesis in pumpkin seedling. **(A)** Proline dehydrogenase (ProDH) activity; **(B)** Δ¹-Pyrroline-5-carboxylate synthase (P5CS) activity; **(C)** Δ¹-Pyrroline-5-carboxylate reductase (P5CR); (D) Ornithine aminotransferase (OAT) activity; **(E)** Glutamate decarboxylase (GAD) activity; **(F)** Betaine aldehyde dehydrogenase (BADH) activity. Data are presented as the mean ± standard deviation (SD) of three replicates. and * indicate significant differences from CK at *P* < 0.05 and *P* < 0.01, respectively. CK, control; GABA, γ-aminobutyric acid.

The ProDH, P5CS, and OAT are key enzymes that catalyze the conversion of Pro, Glu, and Orn to P5C, respectively. Exogenous GABA treatment significantly affected the activities of multiple enzymes in the 2-AP biosynthetic pathway of pumpkin leaves, though the response patterns and magnitudes varied among enzymes. GABA application significantly enhanced ProDH activity, which increased by 51.5% to 770.8% compared to CK during the experimental period (**Figure 4A**). P5CS activity exhibited a multiphasic fluctuation pattern. While GABA treatment did not alter this overall trend, it substantially elevated the enzymatic activity, with increases of 101.1%, 212.9%, and 47.1% compared to CK at 18 h, 30 h, and 48 h, respectively (**Figure 4B**). In contrast, OAT and P5CR activities were significantly suppressed by GABA treatment (**Figures 4C, D**). At 36 h, OAT and P5CR activities in GABA-treated leaves were 13.2% and 16.8% lower than those in CK, respectively.

GAD and betaine aldehyde dehydrogenase (BADH) are key enzymes linking GABA metabolism with 2-AP biosynthesis. GAD activity showed an initial increase followed by a decline in CK. Although GABA treatment did not change the overall pattern, it shifted the peak of GAD activity from 30 h to 24 h. At 18 h, 24 h, and 36 h, the GAD activities in the GABA-treated leaves showed significant increases of 27.8%, 41.5%, and 36.8%, respectively, compared to CK (**Figure 4E**). BADH activity displayed a pattern of initial increase followed by decrease. GABA treatment maintained this dynamic pattern but significantly inhibited the enzyme activity, with reductions of 26.0%, 42.7%, 22.6%, 50.7%, and 9.1% at 12 h, 18 h, 24 h, 30 h, and 48 h, respectively, compared to CK (**Figure 4F**). In summary, exogenous GABA treatment coordinately regulated the metabolic flux toward 2-AP biosynthesis by enhancing the activities of ProDH, P5CS, and GAD while suppressing those of OAT, P5CR, and BADH.

### 3.5 Regulation of key 2-AP biosynthetic gene expression by exogenous GABA

**Figure 5:**
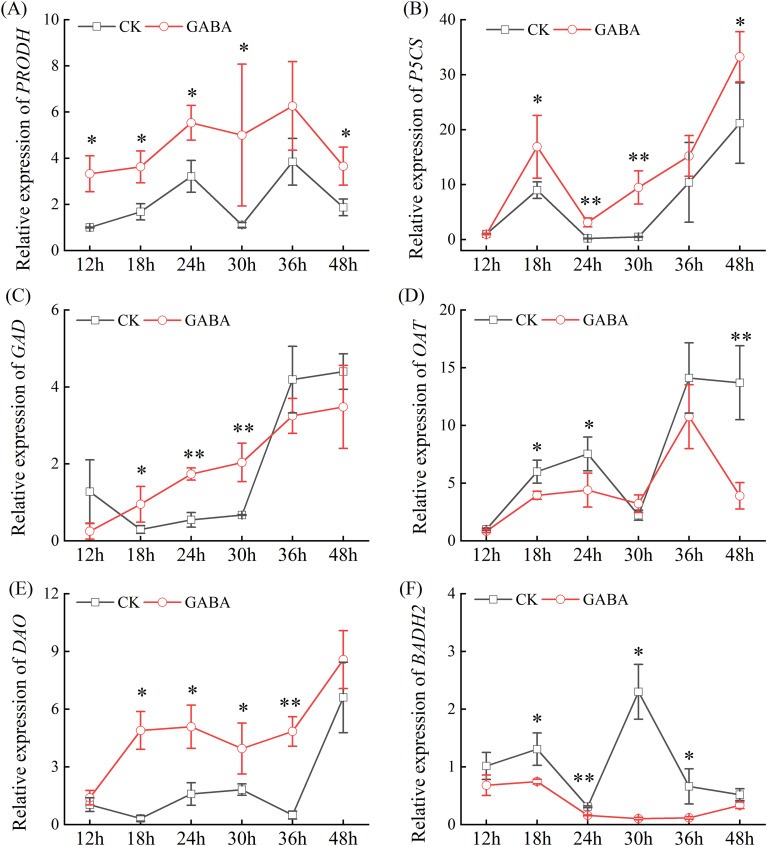
Effects of GABA on the expression of key enzyme genes involved in 2-AP biosynthesis in pumpkin seedling. **(A)** Relative expression of *PRODH*; **(B)** Relative expression of *P5CS*; **(C)** Relative expression of *GAD*; **(D)** Relative expression of *OAT*; **(E)** Relative expression of *DAO*; **(F)** Relative expression of *BADH*. Data are presented as the mean ± standard deviation (SD) of three replicates. * and ** indicate significant differences from CK at *P* < 0.05 and *P* < 0.01, respectively. CK, control; GABA, γ-aminobutyric acid; *PRODH*, proline dehydrogenase; *P5CS*, Δ^1^-pyrroline-5-carboxylate synthase; *GAD*, glutamate decarboxylase; *OAT*, ornithine aminotransferase; *DAO*, diamine oxidase; *BADH*, betaine aldehyde dehydrogenase.

To elucidate the molecular mechanisms underlying GABA-mediated regulation of 2-AP biosynthesis, we analyzed the transcript levels of key structural genes in the pathway, including *PRODH* (*CmoCh15G002000*), *P5CS* (*CmoCh14G001150*), *OAT* (*CmoCh18G004780*), *GAD* (*CmoCh15G008720*), *BADH2* (*CmoCh10G001620*), and *DAO* (*CmoCh10G001000*), using RT-qPCR (**Figure 5**). GABA treatment markedly reshaped the transcriptional landscape, upregulating *P5CS*, *PRODH*, *DAO*, and *GAD* while suppressing *OAT* and *BADH2* expression.

The temporal expression patterns revealed distinct regulatory modes. For *PRODH* (**Figure 5A**) and *P5CS* (**Figure 5B**), GABA treatment preserved their fluctuation patterns but substantially amplified transcript accumulation. During the 12–48 h period, GABA treatment increased *PRODH* expression by 0.7- to 3.5-fold, while it enhanced *P5CS* transcripts by 14.6- and 18.4-fold at 24 h and 30 h, respectively. *GAD* transcription displayed complex response, with GABA treatment altering both expression levels and temporal pattern (**Figure 5C**). Significant upregulation (2.0~2.2 fold) occurred at 18~30 h, while suppression was observed at other time points. Notably, GABA exhibited distinct effects on branch-point enzymes. *OAT* transcription was significantly suppressed by GABA despite maintaining its fluctuating pattern (**Figure 5D**), with reductions of 23.8% and 71.5% at 36 h and 48 h, respectively. Conversely, *DAO* expression was strongly upregulated by GABA, with its expression levels increasing 1.2~14.0-fold compared to CK during 18~36 h period, respectively (**Figure 5E**). Most strikingly, GABA altered the expression pattern of *BADH2*, completely suppressing the transcriptional level of *BADH2* (**Figure 5F**). For example, at 30 h, the expression level of *BADH2* in the GABA-treated group was reduced by 95.5% compared to that in the CK. These results indicated that GABA coordinated the biosynthesis of 2-AP through multifaceted transcriptional regulation: enhancing precursor supply via P5CS and PRODH, promoting the conversion between metabolites through GAD and DAO regulation, while simultaneously eliminating metabolic competition by strongly inhibiting BADH2.

## 4 Discussion

Aroma quality is an essential attribute of pumpkin flavor that significantly influences consumer preference (Junxing et al., 2022). 2-AP has been identified as the principal aroma compound responsible for the characteristic fragrance in Xiangyu pumpkin (Deng et al., 2023), with uniquely flavored varieties commanding greater market acceptance. Previous studies have demonstrated GABA’s efficacy in enhancing 2-AP accumulation in fragrant rice (Gao et al., 2020; Xie et al., 2020). This study investigated the regulatory role of exogenous GABA on 2-AP biosynthesis and its metabolic pathway in pumpkin seedlings. Our findings reveal that GABA promotes 2-AP synthesis in pumpkin primarily through enhancing glutamate metabolism, while concurrently stimulating seedling growth.

### 4.1 Exogenous GABA promotes seedling growth and 2-AP biosynthesis in pumpkin

GABA, a non-protein amino acid, serves as an important signaling molecule involved in plant growth and stress responses (Fait et al., 2008; Ramesh et al., 2016). For instance, under drought conditions, elevated GABA levels in tobacco leaves have been shown to enhance plant adaptability and tolerance (Pelvan et al., 2021). Similarly, exogenous GABA application alleviated salt stress damage in pepper seedlings (AL-Quraan et al., 2024). In this study, we demonstrated that GABA treatment significantly improved plant height, stem diameter, leaf area, and biomass (both fresh and dry weight) in pumpkin seedlings (**Figure 2**), consistent with previous reports in maize (Li et al., 2016) and rice (Xie et al., 2019). Notably, unlike its well-documented role in stress conditions, GABA exhibited significant growth-promoting effects under normal growth conditions, suggesting that GABA functions not only as a stress-responsive metabolite but also as a broad-spectrum physiological regulator. These improvements in growth parameters may be attributed to GABA-mediated regulation of photosynthesis and carbon-nitrogen metabolism (Jin et al., 2023), though the underlying molecular mechanisms in pumpkin remain to be fully elucidated.

Accumulating evidence indicates that GABA plays multiple roles in improving crop nutritional value and flavor quality. For example, exogenous GABA improved apple fruit quality by regulating polyamine metabolism and increasing sugar-acid ratio (Li et al., 2021; Cheng et al., 2023). In edamame, GABA treatment promoted the accumulation of anthocyanins and organic acids, thereby significantly improving nutritional quality (Yu et al., 2022). Furthermore, Xie et al. (2019) reported that GABA application at the panicle initiation stage effectively regulated aroma formation in three fragrant rice varieties. Consistent with these findings, our results demonstrated that an appropriate concentration (500 mg L⁻¹) of GABA significantly increased 2−AP content in pumpkin seedlings (**Figure 1**). The observed decline in 2-AP content at 800 mg L⁻¹ GABA, relative to the optimal 500 mg L⁻¹ treatment, reflects a typical hormesis effect commonly observed with plant hormone and biostimulants (Vargas-Hernandez et al., 2017). This concentration-dependent response underscores the importance of optimizing GABA application rates for maximizing aroma quality without inducing negative physiological effects. These findings across diverse species demonstrate GABA’s conserved role in flavor enhancement across diverse species and supporting its broader application as a flavor quality regulator.

### 4.2 Directional regulation of the metabolic flux of 2-AP synthesis precursors by GABA

The biosynthesis of 2-AP is governed by a complex metabolic network involving multiple precursors and key enzymes. This study systematically revealed the comprehensive regulation of exogenous GABA on the metabolic network in pumpkin. In contrast to the findings of Xie et al. (2020) in rice, where GABA increased Pro content, GABA treatment in pumpkin reduced both Pro and Orn levels while significantly enhancing Glu accumulation (**Figure 3**). This species-specific difference likely stems from distinct metabolic partitioning: in pumpkin, GABA appears to preferentially channel Glu into the 2-AP biosynthetic pathway rather than toward Pro synthesis or accumulation. At enzymatic and transcriptional levels, GABA treatment markedly enhanced the activity and expression of P5CS and ProDH (**Figure 4**, **5**), indicating a promoted conversion of Glu and Pro to the intermediate metabolite P5C. These findings were in line with reports that 2-AP synthesis in fragrant rice is promoted by elevated P5CS activity through zinc application (Luo et al., 2019) or by enhanced ProDH activity via nitrogen management (Deng et al., 2018). Conversely, GABA suppressed both the activity and gene expression of P5CR, a key enzyme that catalyzes the reduction of P5C back to Pro (Ma et al., 2025). Inhibiting P5CR effectively blocks the reflux of P5C toward Pro, thereby redirecting metabolic flux toward 2-AP synthesis instead of Pro accumulation (Bao et al., 2023). Together, these findings demonstrate that GABA achieves precise regulation of the pivotal node P5C through a dual strategy, promoting forward synthesis while inhibiting reverse reduction, ultimately enhancing 2-AP biosynthesis.

### 4.3 GABA enhances the 2-AP biosynthetic pathway through coordinated regulation of key genes

The regulatory role of GABA in 2-AP biosynthesis encompasses not only precursor metabolism but also transcriptional reprogramming of multiple key genes. This study found a strong inhibitory effect of GABA on the BADH activity and *BADH2* gene expression in pumpkins (**Figures 4F**, **5E**). BADH serves as a key negative regulator of 2-AP biosynthesis, catalyzing the oxidation of GABald to GABA, thereby competitively consuming Δ¹-pyrroline, the direct precursor of 2-AP (Wakte et al., 2017; Banerjee et al., 2020). Classical studies in rice have established that loss-of-function mutations in *BADH2* represent the primary genetic basis for aroma formation in fragrant rice varieties (Giang et al., 2023). Similarly, knockout of *GmBADH2* successfully induced 2-AP synthesis in soybean (Arikit et al., 2010). Our physiological experiments demonstrate that exogenous GABA treatment similarly suppresses BADH at both transcriptional and enzymatic levels, thereby relieving its inhibitory effect on 2-AP accumulation. However, the precise molecular mechanism by which GABA regulates *BADH2* transcription remains unknown. Future studies should focus on identifying the transcription factors or cis-elements involved in this regulatory pathway.

Concurrently, GABA treatment significantly enhanced the gene expression and enzymatic activities of both GAD and DAO (**Figures 4**, **5**). GAD, as the key enzyme in GABA synthesis, enhances the conversion of Glu to endogenous GABA when upregulated, while increased DAO expression potentially promotes GABald production through the polyamine degradation pathway (Yu et al., 2022). The enhancement of these two processes synergistically elevates the potential supply of GABald. Against the background of significantly inhibited BADH2 activity, the elevated GABald pool is more likely to undergo non-enzymatic cyclization into Δ¹-pyrroline, which subsequently contributes to 2-AP synthesis. Therefore, exogenous GABA effectively optimizes the aroma synthesis network in pumpkin through a dual strategy: enhancing precursor supply while suppressing competitive metabolic pathways. This coordinated regulation at multiple nodal points represents an efficient mechanism for enhancing 2-AP production in pumpkin.

## 5 Conclusion

**Figure 6:**
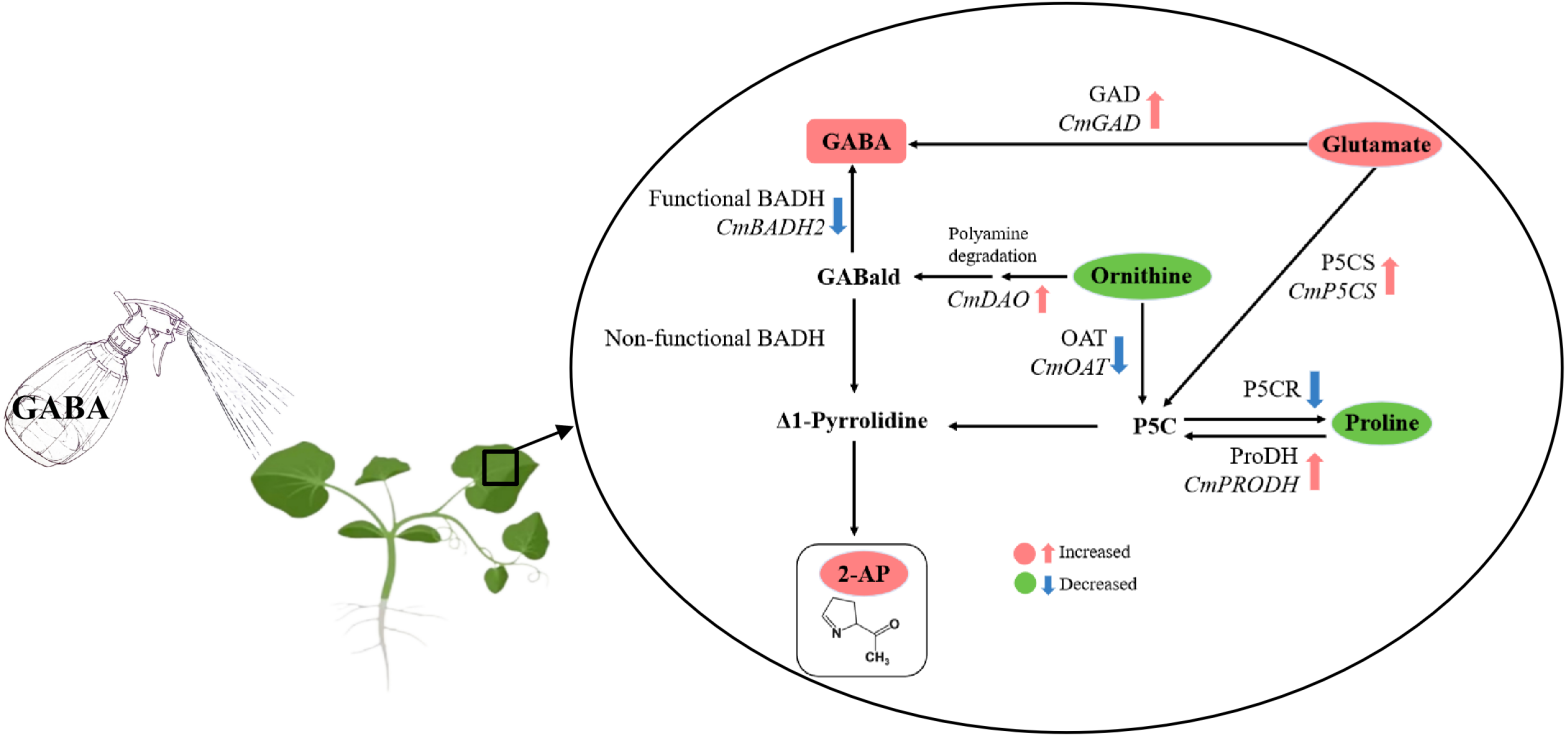
Potential pathways of GABA influencing the biosynthesis of 2-AP in pumpkin. 2-AP, 2-acetyl-1-pyrroline; BADH, betaine aldehyde dehydrogenase; GABA, γ-aminobutyric acid; GABald, γ-aminobutyraldehyde; GAD, glutamate decarboxylase; Glu, glutamate; OAT, ornithine aminotransferase; Orn, ornithine; P5C, pyrroline-5-carboxylate; P5CR, Δ¹-pyrroline-5-carboxylate reductase; P5CS, Δ¹-pyrroline-5-carboxylate synthase; Pro, proline; ProDH, proline dehydrogenase. The upright font represents enzymes, and the italic font represents genes. Upright font indicates enzyme and italic font indicates gene.

This study demonstrates that exogenous GABA serves as an effective biostimulant to enhance aroma quality in pumpkin, and elucidates its mechanism of action from the perspectives of metabolite partitioning and gene expression regulation (**Figure 6**). Foliar application of GABA significantly promoted the accumulation of the key aroma compound 2-AP in “Xiangyuanzao” pumpkin leaves, while simultaneously modulating the metabolism of precursor amino acids, as reflected by a marked increase in Glu content and decreases in Pro and Orn levels. GABA treatment significantly enhanced the activities and gene expression of ProDH and P5CS, while simultaneously suppressing the enzymatic activities of P5CR, OAT, and BADH as well as the transcript level of *BADH2*, thereby coordinately promoting 2-AP biosynthesis. Furthermore, exogenous GABA exhibited beneficial effects on the growth of pumpkin seedling. These findings provide a novel strategy for the chemical regulation of crop flavor quality. Future research should focus on integrating GABA application with optimized water and nutrient management practices to explore its potential in high-efficiency and quality-oriented pumpkin production.

## Data Availability

The original contributions presented in the study are included in the article/**Supplementary Material**. Further inquiries can be directed to the corresponding author.
