# Supplementary material for: Exogenous γ-aminobutyric acid promotes 2-acetyl-1-pyrroline biosynthesis by enriching glutamate and optimizing its metabolic flux in pumpkin
Source: Front Plant Sci. 2026 Apr 10;17:1808004. doi: 10.3389/fpls.2026.1808004 (PMC13106422; doi:10.3389/fpls.2026.1808004)
Supplement: Supplementary file 1 [file DataSheet1.docx]

Table S1 Sequence of gene primers for enzymes involved in 2-AP biosynthesis

| **Gene name** | **Gene ID** | **Forward primer sequence (5’-3’)** | **Reverse primer sequence (5’-3’)** | **Description** |
| --- | --- | --- | --- | --- |
| *Actin* | *CmoCh11G015080* | CGGCCATTGAGAAAAGCTACGAAC | CCCACCACTGAGGACGATGTTACC | Internal gene |
| *PRODH* | *CmoCh15G002000* | TCTGCCAAAGTTGCGTGGATGC | ATGATGGCGGCGGAGTATGTGA | Proline dehydrogenase |
| *P5CS* | *CmoCh14G001150* | TGTGCTGGCATTCCTGTTGTCA | TGAAGCCGCCTTGAACTTTCCC | Delta-1-pyrroline-5-carboxylate synthase |
| *OAT* | *CmoCh18G004780* | TCTTCCCGTTGGAGCCGTACTA | AGCATTGCAGACGAGTGGTGAG | Ornithine aminotransferase |
| *GAD* | *CmoCh15G008720* | ACCGAAGTACCCACGACGAGTT | ACACGAAGAACCGACACATGCT | Glutamate decarboxylase |
| *DAO* | *CmoCh10G001000* | TGCGGAATGTGAGGCTGTTGTC | AGCAAGTCTTCGACCAGGAGCA | Diamine oxidase |
| *BADH2* | *CmoCh10G001620* | TGCTGCTAAGGTGAGTCGGAGT | GCATCCAACCCTTCGGCAAGAT | Betaine aldehyde dehydrogenase |


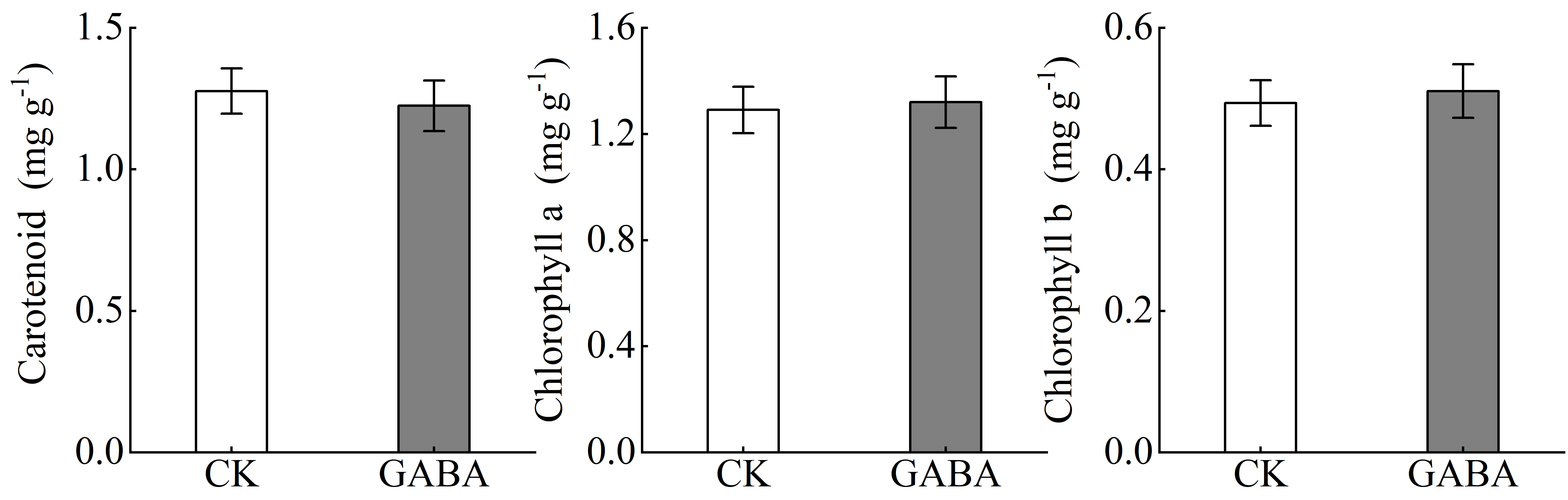


Figure S1 Effects of γ-aminobutyric acid (GABA) on the pigment content of “Xiangyuanzao” pumpkin seedlings.
